# Remote-Customized Telecontrol for Patients with Rheumatoid Arthritis: The iARPlus (Innovative Approach in Rheumatology) Initiative

**DOI:** 10.3390/jpm15010030

**Published:** 2025-01-16

**Authors:** Fausto Salaffi, Sonia Farah, Eleonora Di Donato, Massimo Sonnati, Emilio Filippucci, Rossella De Angelis, Francesco Gabbrielli, Marco Di Carlo

**Affiliations:** 1 Rheumatology Unit, Università Politecnica delle Marche, “Carlo Urbani” Hospital, Via Aldo Moro, 25, 60035 Jesi, Italy; fausto.salaffi@gmail.com (F.S.); sonia.farah91@gmail.com (S.F.); eleonoradidonato@ymail.com (E.D.D.); emilio_filippucci@yahoo.it (E.F.); r.deangelis@staff.univpm.it (R.D.A.); 2 Hippocrates Sintech Srl, 60035 Genova, Italy; m.sonnati@hippocrates-sintech.it; 3 Lead of R&D for Clinical Activity in Telemedicine, Italian National Agency for Healthcare (Agenas), 60035 Roma, Italy; gabbrielli@agenas.it

**Keywords:** rheumatoid arthritis, telecontrol, RAID, disease activity

## Abstract

**Objective**. Telecontrol approaches for rheumatoid arthritis (RA) management aim to enhance patient outcomes. This pilot study assessed whether the Rheumatoid Arthritis Impact of Disease (RAID) approach could be used during teleconsultations to monitor RA disease activity through a web-based platform called iARPlus (Innovative Approach in Rheumatology). **Methods**. Forty RA patients participated in two in-person visits (baseline and 12 months) and seven teleconsultations over 12 months, collected via the iARPlus portal and accessible through an internet browser. Disease activity, at baseline and follow-up, was measured using the Clinical Disease Activity Index (CDAI) and self-reported RAID scores throughout the study. The RAID approach, developed by the European Alliance of Associations for Rheumatology (EULAR), combines key patient-reported outcomes (PROs). **Results**. Nineteen patients (mean age: 49.3 years) were treated with Janus kinase inhibitors (JAKis), and 21 patients (mean age: 48.1 years) received adalimumab. All patients had active disease (mean CDAI 27.9 ± 4.8). Strong correlations were found between CDAI and RAID scores at baseline (ρ = 0.809, *p* < 0.0001) and at follow-up (ρ = 0.789, *p* < 0.0001). JAKi-treated patients showed greater reductions in RAID scores, pain relief, and higher rates of disease remission compared to adalimumab-treated patients. **Conclusions**. RAID scores were effective in teleconsultations for assessing RA disease activity. JAKi treatment resulted in better pain control and disease activity improvement compared to adalimumab. Further studies are needed to confirm the clinical and economic benefits of telecontrol for RA management.

## 1. Introduction

Rheumatoid arthritis (RA) is a chronic inflammatory joint disease that affects 0.5–2% of the population [1]. Its progression is unpredictable, with a wide range of severity. The management of RA in clinical practice has significantly improved with the introduction of synthetic and biological disease-modifying antirheumatic drugs (DMARDs) [2,3]. Janus kinase inhibitors (JAKis) represent the latest class of disease-modifying drugs for RA treatment [4,5]. These small molecule-targeted therapies are the first oral options to demonstrate comparable efficacy to existing biologic DMARDs. Tofacitinib, baricitinib, upadacitinib, and filgotinib are the JAKis currently available. All these innovative drugs have demonstrated efficacy in RA patients with active disease who have shown an inadequate response to conventional synthetic DMARDs [6,7]. In 2019 and 2023, JAKis were recommended as second-line treatments for RA, with efficacy and safety levels comparable to biological DMARDs [2,8]. JAKis are effective in patients with difficult-to-treat RA, even in those who have previously been treated with at least two biological DMARDs [9,10].

Numerous clinical studies have demonstrated the benefits of strict disease control, including regular monitoring and therapy escalation based on a target level of disease activity [11]. This approach, aiming for minimal disease activity or remission, appears to be more critical than the specific medication used [11,12]. Effective interventions reduce hospitalization rates and improve patient outcomes. The level of disease activity achieved is a crucial predictor of future disability and radiographic progression [13]. Consequently, the “treat-to-target” (T2T) strategy has become an attractive concept in RA management. Although Italian rheumatologists are generally well informed and supportive of T2T recommendations for RA [14], several barriers hinder the widespread adoption of this approach.

Firstly, managing DMARDs can be challenging for non-rheumatologists, and rheumatologists are not always easily accessible to RA patients. Secondly, even when rheumatologist access is available, many patients may not opt for a T2T strategy. Thirdly, rheumatologists with busy practices may face difficulties in implementing T2T, as it requires frequent visits and the use of systematic joint counts for standardized RA disease activity assessments [15]. These assessments, including swollen joint counts (SJCs) and tender joint counts (TJCs), are time-consuming, and few rheumatologists have the time to incorporate them into their routine practice [16,17,18].

The growing interest in tracking disease progression and therapeutic response has led to the increased use of various patient-reported outcomes (PROs). These measures aim to improve care and screen for physical or psychosocial issues in routine clinical practice, clinical trials, and long-term clinical registries [19,20,21,22]. The rising importance of PROs is for enhancing the delivery of accurate and timely medical information while streamlining processes to expedite patient care. However, the challenge of integrating the administration and analysis of PROs into standard clinical practice remains a significant barrier to their routine collection.

The European Alliance of Associations for Rheumatology (EULAR) developed the Rheumatoid Arthritis Impact of Disease (RAID) questionnaire, a score that reflects RA disease activity from the patient’s perspective [23,24,25]. The RAID method has proven highly responsive to changes in RA patients with short disease duration who followed a T2T strategy. It is also a valuable tool for teleconsultations, showing a strong correlation with disease activity measures in this setting [26,27]. During teleconsultations, a RAID score threshold of two can distinguish patients with good disease control from those who may require an in-person visit [28]. However, administering, scoring, and interpreting PROs can be challenging and often require manual data computation, which is both error-prone and time-consuming. To address these challenges, researchers are developing and evaluating alternatives to traditional paper-based instruments [29,30,31,32,33,34,35].

Advancements in interactive computer technology have provided patients with greater opportunities to participate in the design, execution, and assessment of their care. Various platforms can now be used to evaluate PROs, including touch-screen computers in offices [21,22], telephone-based interactive voice-response (IVR) systems [31,32], handheld computers [36], mobile phones [31,32], and internet-based approaches [31]. Each of these technologies supports routine PROs collection and facilitates the shift from institution-centric to patient-centric care approaches [30,31,33,37,38,39].

Home telecontrol is a relatively new and increasingly used method [34]. It is the most promising application of telecontrol technology for providing high-quality, cost-effective care [29]. Home telecontrol is a fundamental component of a broader perspective on deinstitutionalization, reflecting a societal shift towards keeping patients in their homes [35,38]. Repeated, personalized, and dosed information should enhance patients’ disease-specific knowledge and self-care skills, thereby increasing their self-efficacy and improving adherence. Published telemonitoring studies have reported limited findings on the effects of knowledge and self-care [39,40,41], self-efficacy [35], or therapy adherence [42,43,44].

Starting from these assumptions, the objective of this study is to assess the applicability of the RAID method, as a disease activity measure, in a teleconsultation setting by using a web-based system called Innovative Approach in Rheumatology (iARPlus).

## 2. Methods

### 2.1. Study Population

This prospective pilot study included 40 patients diagnosed with RA according to the 2010 American College of Rheumatology (ACR)/EULAR criteria [45]. All patients were on methotrexate (MTX) for ≥3 months at a stable dose of 15 mg/week for ≥4 weeks prior to the first dose of the study drug. Patients exhibited active disease, defined as a Clinical Disease Activity Index (CDAI) > 22 [46]. The disease duration was calculated from the onset of symptoms to the baseline, which coincided with the point of diagnosis and the initiation of treatment. Patient selection was conducted at the outpatient clinic by two rheumatologists (FS and MDC).

Patients were administered with one of the following treatments: upadacitinib at 15 mg QD, filgotinib at 200 mg QD, baricitinib at 4 mg QD, or adalimumab at 40 mg EOW, while continuing MTX therapy. They were also permitted to remain on oral glucocorticoids (GCs). MTX was co-prescribed with 10 mg/week of folic acid, taken two days after the MTX dose.

Both upadacitinib and filgotinib preferentially inhibit JAK1 activity. These small molecules have demonstrated efficacy in treating RA across various populations, leading to significant improvements in RA symptoms, a reduction in radiographic progression, and positive impacts on PROs such as pain and quality of life [47,48,49,50].

Exclusion criteria included patients who were unable to provide informed consent, those with visual or auditory impairments who lived alone, individuals lacking fluency in Italian, patients with chronic obstructive pulmonary disease (COPD), ischemic heart disease, valvular disorders, heart failure or stroke within the previous 2 years, multiple sclerosis, extracorporeal dialysis, chronic infectious diseases, or patients who required hospitalization or treatment with intravenous antibiotics within 30 days or oral antibiotics within 14 days prior to screening. Additional exclusion criteria encompassed concurrent treatment with experimental drugs, malignancy within the last 10 years, cytopenia (hemoglobin < 9 g/dL in men and <8.5 g/dL in women, leukocytes < 3 × 10^9^/L, and platelets < 150 × 10^9^/L), serum aspartate aminotransferase/alanine aminotransferase levels > 1.5 times the upper limit of normal, creatinine clearance < 50 mL/min, pregnancy, inadequate contraception, alcohol abuse, and psychological or cognitive impairments that would prevent adherence to the study protocol. Patients at high risk for tuberculosis were allowed to participate after completing chemoprophylaxis, as recommended in Italy [51].

The study was conducted in accordance with the principles outlined in the 1964 Declaration of Helsinki and its later amendments and was approved by the local ethics committee (Comitato Etico Unico Regionale, n. 169-2019, date of approval: 5 May 2019).

### 2.2. Variables

Rheumatoid factor (RF) and anti-citrullinated protein autoantibody (ACPA) statuses were determined at baseline. The CDAI was assessed at baseline and after 12 months. The CDAI excludes C-reactive protein (CRP) levels and is calculated by summing the counts of swollen and tender joints (28-joint count) along with the evaluator’s and/or patient’s global assessment of disease activity (EGA and PGA) using a numerical rating scale (NRS) ranging from 0 to 10 cm [50,51,52]. CDAI values can range from 0 to 76. High disease activity (HDA) is defined as a CDAI > 22, moderate disease activity (MDA) as a CDAI > 10 and ≤22, low disease activity (LDA) as a CDAI > 2.8 and ≤10, and remission (REM) as a CDAI ≤ 2.8 [52,53,54,55].

The validity of the CDAI was established by Aletaha et al., who assessed its correlational validity (i.e., its comparison with other measures of disease activity), discriminant validity (i.e., its correlation with changes in other measures of disease activity), and construct validity (i.e., its correlation with important disease outcomes, such as radiological progression), using various statistical methods [50,51,52]. A major advantage of the CDAI is its ease of use for frequent, consistent patient assessments without requiring a calculating device. Additionally, the CDAI cutoff values for remission are more stringent than those of the DAS28, allowing for less residual disease activity.

The RAID method was developed as an initiative of the EULAR to combine the most important PROs into a single measurement. This patient-centered index was designed for clinical trials in RA to replace the use of multiple questionnaires without losing critical information and to assess changes in disease outcomes over time. The RAID score includes seven domains: pain, function, fatigue, physical and psychological well-being, sleep disturbance, and coping [23,24].

Each RAID domain is measured on a numerical rating scale from 0 (best) to 10 (worst), with specific weights assigned to each domain in the final sum score: pain (21%), functional disability (16%), and fatigue (15%) as well as sleep disturbance, physical well-being, psychological well-being, and coping (12% each). An absolute or relative minimal clinically important improvement (MCII) of at least 3 points or more than 50% improvement was proposed, along with a patient acceptable symptom state (PASS) score of ≤2 [56]. Suggested cut-off values for disease impact are as follows: RAID ≤ 3 (remission), RAID > 3 and ≤4 (low impact of disease), RAID > 4 and ≤6 (moderate impact of disease), and RAID > 6 (high impact of disease) [26].

Avouac et al. found the RAID score effective as a triage tool to identify patients requiring expedited in-person clinic review and as a tool in telemedicine settings [28].

### 2.3. Self Administration of the RAID via Web

All the patients enrolled in this study participated in two in-person visits (at baseline and 12 months of follow-up) and seven remote visits (at 1, 2, 3, 4, 5, 6, and 9 months). The timing of the visits was determined using a treat-to-target approach, with monthly assessments during the first six months, followed by quarterly evaluations thereafter. The web portal, accessible to authorized users via personal computers and internet browsers, offers a user-friendly graphical interface designed for easy navigation (https://www.iarplus.it/login, accessed on 12 December 2024). Responses were recorded by selecting one of the radio buttons on the screen, with each question requiring completion before proceeding to the next. The interface was presented in Italian. Upon registration, each patient was provided with a secure username and password to access the service through the web portal.

Prior to completing the computerized questionnaires, all patients participated in a brief information/training session to familiarize them with the PC’s components and technical aspects of the response process. The wording of the computerized system’s questions was identical to that of the paper-and-pencil format. No prior information was provided, but an instructor (SF) was available for on-demand tutoring, and technical support was accessible via telephone or email throughout the study. Data entry was performed using a template by selecting the appropriate box, followed by data processing to generate statistics and trends.

The iARPlus system recorded the following patient variables: demographic data, disease duration, and responses to the RAID questionnaire. Additionally, the frequency of web portal logins was tracked through a monthly questionnaire over the 12-month period.

At the conclusion of the study, the electronically collected raw data, including patient numbers, age, gender, duration of assessments, and test results, were extracted from the practice computers and pseudonymized. This information was made available to the telemedicine clinical team.

The iARPlus system is secure and complies with the Health Insurance Portability and Accountability Act (HIPAA) regulations, employing highly encrypted formats for remote database storage and secured transmissions via the Secure Sockets Layer (SSL) communication protocol.

During remote monitoring, if the RAID scores indicated a phase of high disease impact (RAID > 6), patients were recalled for therapeutic adjustments concerning the dosage of oral corticosteroids. In the case of persistent mono- or oligo-articular inflammatory symptoms, patients could be recalled and scheduled for intra-articular corticosteroid injections.

### 2.4. Statistical Analysis

For the analyses conducted in this study, patients were divided into two groups: those undergoing treatment with JAKis and those receiving treatment with adalimumab.

Descriptive statistics for continuous data are reported as mean (SD) and median (interquartile range, IQR), while categorical data are expressed as percentages. The cumulative disease activity burdens, as measured by the RAID score from baseline to 52 weeks, the percentage of patients achieving CDAI REM, and the percentage of patients reaching CDAI LDA, were utilized as effectiveness measures in this analysis. Over the one-year follow-up period, time-integrated metrics, including the Last-First difference, percentage change in Last-First values, and area under the curve (AUC), were calculated for each patient to quantify the cumulative inflammatory load.

The Mann–Whitney U test or Student’s *t*-test was employed to compare continuous data between the two groups. A chi-square test with continuity correction was used to compare differences in CDAI response thresholds between the treatment groups. Univariate analysis and Spearman’s correlation coefficient were applied to evaluate correlations between CDAI and RAID scores at baseline and after 24 weeks. Receiver operating characteristic (ROC) curve analysis, with the Youden index (J), was used to assess the discriminatory value of the RAID score, analyzing the AUC and corresponding ROC curve coordinates. All statistical analyses were performed using MedCalc version 19.5 (Mariakerke, Belgium).

## 3. Results

The study population included nineteen patients in group 1, treated with JAKis (nine patients on upadacitinib, five on filgotinib, and five on baricitinib), and twenty-one patients in group 2, treated with adalimumab. All patients had active disease, with a mean (SD) CDAI of 27.9 (4.9). Of the total patients, 31 (77.5%) were RF positive and 27 (67.5%) were ACPA positive. Additionally, 17 patients (42.5%) across both groups were on oral steroids.

There were no statistically significant differences in demographic and baseline disease characteristics between the two groups (Table 1). Specifically, the total score and subdomains of the RAID were comparable between the groups.

**Figure 1:**
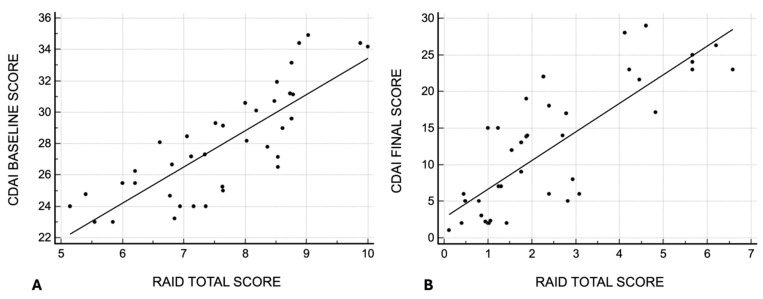
Correlation between the CDAI total score and the RAID total score at baseline (**A**) and at 12 months of the follow-up (**B**).

A significant correlation was observed between CDAI scores and RAID scores, both at baseline (rho = 0.809, *p* < 0.0001) and at the end of the follow-up (rho = 0.789, *p* < 0.0001) consultations (Figure 1).

Significant differences were found between the JAKis and adalimumab groups when comparing the Last-First values (adalimumab vs. JAKis: −4.248 ± 2.196 vs. −6.828 ± 1.495; *p* = 0.034), percentage difference in Last-First values (adalimumab vs. JAKi: −58.528 ± 28.954 vs. −76.591 ± 16.270; *p* = 0.041), and the AUC of the RAID scores (RAID-AUCs) (adalimumab vs. JAKi: 41.142 ± 12.377 vs. 32.152 ± 9.842; *p* = 0.016) (Table 2).

**Figure 2:**
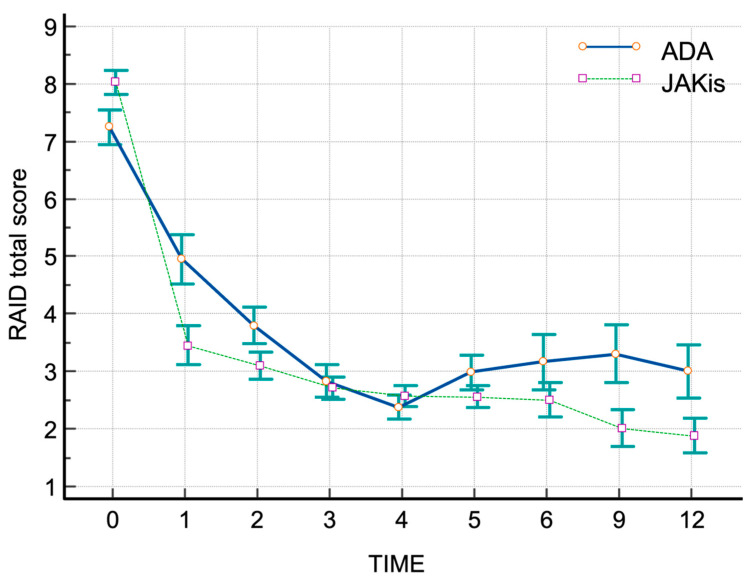
Plot of trajectories for RAID total score across visits. Mean scores (±SEM) of disease activity, as measured with the time integration (AUC) plot and the RAID total scores at 0-, 1-, 2-, 3-, 4-, 5-, 6-, 9-, and 12-month intervals in JAKis (dotted line) and adalimumab (solid line) groups.

**Figure 3:**
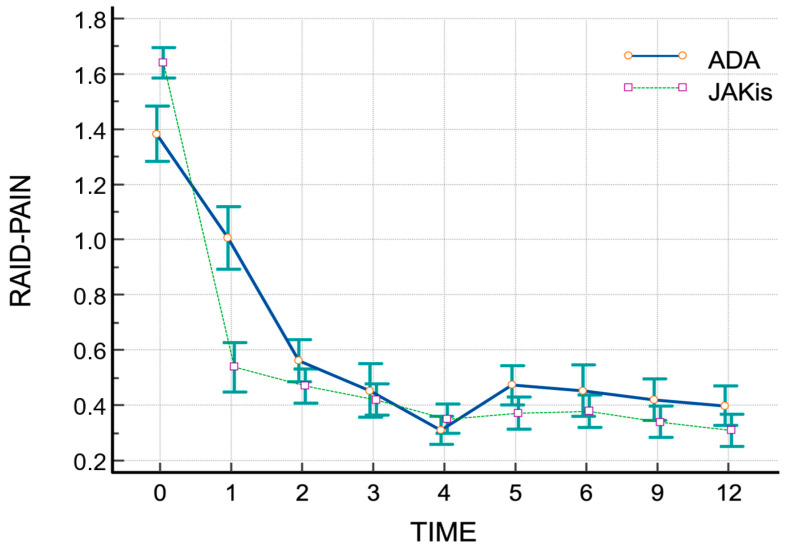
Plot of individual trajectories for RAID-PAIN scores across visits. Mean scores (±SEM) of pain, as measured via the time integration (AUC) plot and the RAID-PAIN scores at 0, 1, 2, 3, 4, 5, 6, 9, and 12-month intervals in JAKis (dotted line) and adalimumab (solid line) groups.

Plots of RAID score trajectories at 0-, 1-, 2-, 3-, 4-, 5-, 6-, 9-, and 12-month intervals for both groups are shown in Figure 2. Patients treated with JAKis demonstrated greater pain relief at one month compared to those on adalimumab (treatment difference: −12.3, 95% CI −17.9 to −6.6) (Figure 3). A higher proportion of JAKis-treated patients achieved CDAI REM or CDAI LDA compared to adalimumab-treated patients (23.8% vs. 10.5% at year 1, *p* < 0.01 for remission, and 33.3% vs. 21.0%, *p* < 0.05 for low disease activity at year 1).

A RAID score of 3.59 (rounded off to 4) demonstrated high sensitivity (89.47%) in predicting CDAI REM or LDA, assuming inadequately controlled disease activity for higher values (Table 3).

## 4. Discussion

RA is a condition that imposes significant burdens on patients due to pain and functional limitations [26,57]. Evaluating the effectiveness of treatments for chronic pain conditions like RA necessitates prioritizing patients’ values and perspectives. PROs are essential in this regard, as they provide subjective evaluations of disease impact [20,21,22,26,58,59]. Patients’ perspectives on disease outcomes may differ from those of their physicians, and significant patient–physician discordance has been linked to negative health outcomes, including medication non-adherence and decreased overall satisfaction [60,61,62,63].

Research has shown that PROs, compared to physician-reported outcomes, offer better discrimination of treatment effects on symptoms [58,64,65]. The RAID method meets several crucial criteria: it is feasible; it is patient-derived, and it provides both a comprehensive profile and a numerical score [22,23,24,25,26,28]. These features are pivotal for an instrument to be used for telemedicine consultation.

Chronic diseases like RA negatively affect both the lives of patients and their caregivers [64,65,66,67,68]. High-quality care for chronic conditions is characterized by positive interactions with caregivers, regular assessments, support for self-management, and therapy optimization, all of which contribute to improved clinical and patient outcomes. Wagner et al. observed that such interactions do not necessarily require face-to-face visits [69].

Telecontrol could serve as a tool for shared decision-making by providing an additional source of information to educate RA patients about the prognosis, treatment options, typical symptoms, and disease etiology [70]. Shared decision-making emphasizes the patient’s role as an active partner with the physician in selecting the best course of action [71,72]. Home telecontrol appears to be a promising strategy for patient management, producing accurate and reliable data, empowering patients, influencing their attitudes and behaviors, and potentially improving their medical conditions, as indicated by a systematic review of experimental and quasi-experimental studies involving chronic patients [73].

A comprehensive review of studies on the acceptability of telemedicine as a substitute for in-person care has found it to be both advantageous and effective, improving treatment outcomes, technological usability, and healthcare efficiency [74]. However, high technical complexity in home-based self-management systems, especially when patient data entry is cumbersome, has been noted in some trials. The lack of perceived immediate benefits is a significant barrier to technology adoption. For patients with RA who already take personal responsibility for their healthcare, such as those with strong medication compliance, telecontrol systems may be perceived as burdensome and intrusive. Tailoring telecontrol programs to patient profiles, considering factors such as education, literacy, comorbidities, disease severity, and stage, is crucial for patient motivation and their effective use. The system must be user-friendly, as complicated systems can be stressful and off-putting. Clarifying the intended benefits and employing easily navigable technology are essential to optimize the utilization of telecontrol in RA care [75]. However, widespread adoption of telecontrol in RA remains limited due to obstacles at both the patient and organizational levels, such as unreliable internet connections and living conditions.

Current recommendations for treating RA emphasize on achieving clinical remission to prevent joint deterioration and improve physical function and quality of life [2,8]. Patients receiving JAKis reported better pain reduction after one month compared to those receiving adalimumab (treatment difference: −12.3, 95% CI −17.9 to −6.6). Both the EULAR and the ACR recognize remission as a crucial metric for predicting improvements in physical function and preventing radiographic progression. For patients who cannot achieve remission, low disease activity is considered a suitable alternative, according to the 2013 and 2019 EULAR updates. A higher proportion of JAKi patients achieved CDAI low disease activity or remission compared to adalimumab patients at one year.

Improving RA patient monitoring during clinical visits and at home is a significant application of telecontrol. Frequent data collection aims to enhance the assessment of patient status, enabling the early detection of health decline and timely intervention to potentially prevent hospitalization. In this study, the cumulative inflammatory load was measured over one year and expressed as time-integrated values (e.g., differences in Last-First values, percentage differences in Last-First, and AUC). The use of AUC, which converts multivariate data into univariate space, simplifies statistical analysis, especially in scenarios with repeated measurements, by reducing the number of statistical comparisons required [76,77,78]. This approach is advantageous compared to repeated measures analysis of variance, which may not account for varying time intervals between measurements. The CDAI and RAID showed a strong correlation at baseline and at the end of follow-up.

JAKis modulate numerous pro-inflammatory cytokines [79,80]. Despite similar efficacy on standard inflammation markers, randomized clinical trials on JAKis in RA have shown greater pain reduction compared to other mechanisms of action [81,82]. The impact of JAKis on pain might be independent of clinical joint swelling [83,84]. Studies compared the efficacy of baricitinib and methotrexate monotherapy in pain management in early RA and found that baricitinib, along with other JAKis, is more effective in advanced RA patients than tumor necrosis factor blockers or interleukin-6 inhibitors [83,85,86]. However, the independent effect of JAKis on pain needs further confirmation beyond clinical trials.

This study represents the first clinical research assessing a customized telecontrol strategy in the Italian RA population. The telecontrol system is based on the premise that increased knowledge about a specific condition leads to better self-care, which subsequently improves clinical outcomes and quality of life. The RAID method, with its strong link to disease activity, is a valuable tool for teleconsultations and can be used as a triage tool to identify patients needing accelerated in-person evaluations. This study confirmed that a RAID score of ≤4 indicates adequate disease control, consistent with previous reports [25,26].

While telecontrol systems for collecting subjective data in rheumatologic settings are practical and can achieve high compliance rates across RA patients, significant barriers include inadequate device usability, insufficient training, low computer literacy, and limited self-efficacy. Healthcare providers, including nurses and clinicians, require specialized training, as providing care via telecontrol demands a specific skill set. The absence of a professional project manager is a significant challenge in integrating telecontrol into routine practice, often leading to overestimations of telehealth activity duration. Some organizations struggle to provide adequate computer access to their telehealth staff, and there is often a lack of awareness among senior management regarding the potential of telemedicine to drive innovation in healthcare delivery. Additionally, interoperability issues, where disparate systems cannot communicate or exchange information, further hinder the adoption of telecontrol. These challenges discourage clinicians from utilizing telecontrol, despite its potential benefits [87,88].

The financial implications of telemedicine are another significant concern. While some evaluations suggest that telemedicine may be cost-effective, the broader social and organizational costs are often underestimated. Short study durations may limit the generalizability of the findings compared to longer time frames, and the rapid evolution of telecontrol technology complicates long-term assessments. The primary goals of telecontrol research are to determine its impact on hospitalization rates, costs, morbidity, mortality, and quality of life.

This study has certain limitations that should be acknowledged. The primary ones include its monocentric design and the small sample size. Additionally, it is important to note that this telemedicine approach is more applicable to a relatively younger population of patients with RA who are proficient with digital technology, and it may not be feasible for older patients.

## 5. Conclusions

Telecontrol systems like iARPlus represent an innovative approach to patient care. However, their successful implementation require changes in both patient and caregiver attitudes as well as organizational structures. Telecontrol must be user-friendly and must integrate seamlessly into the broader care system to be both effective and economical. Additionally, ongoing education for patients and caregivers, along with system evaluations, is necessary to ensure their optimal use. Post-implementation, the outpatient care system and financial frameworks may require reorganization to fully integrate telecontrol into routine care.

## Figures and Tables

**Table 1:** Baseline demographic and disease characteristics.

Variables	Group 1 (JAKis)	Group 2 (ADA)	*p* Value
Number of patients	19	21	-
Age (years), mean ± SD	49.3 ± 15.2	48.1 ± 16.3	0.842
Women, *n* (%)	16 (80.0)	15 (78.9)	0.909
Symptom duration, mean ± SD	3.8 ± 1.3	3.5 ± 1.6	0.543
RF positive, *n* (%)	16 (77.4)	15 (70.0)	0.873
ACPA positive, *n* (%)	14 (58.6)	13 (66.7)	0.971
CDAI, mean ± SD	28.02 ± 5.88	27.91 ± 4.78	0.078
RAID total score, mean ± SD	7.24 ± 1.32	8.02 ± 0.98	0.058
RAID pain, mean ± SD	1.38 ± 0.44	1.64 ± 0.25	0.055
RAID functional disability, mean ± SD	1.19 ± 0.22	1.27 ± 0.16	0.166
RAID fatigue, mean ± SD	1.18 ± 0.24	1.29 ± 0.25	0.138
RAID sleep, mean ± SD	0.69 ± 0.32	0.88 ± 0.26	0.053
RAID physical well-being, mean ± SD	0.89 ± 0.17	0.95 ± 0.12	0.165
RAID emotional well-being, mean ± SD	1.03 ± 0.19	1.02 ± 0.14	0.889
RAID coping, mean ± SD	0.89 ± 0.17	0.95 ± 0.12	0.167

Abbreviations. JAKis = Janus kinase inhibitors; ADA = adalimumab; SD = standard deviation; RF = rheumatoid factor; ACPA = anti-citrullinated protein autoantibody; CDAI = Clinical Disease Activity Index; RAID = Rheumatoid Arthritis Impact of Disease.

**Table 2:** Statistical analysis (Mann–Whitney U test) of the difference Last-First, % difference Last-First, and area under curve values of the RAID method in two groups of patients.

Difference Last-First values						
Group	n	mean	95% CI	SD	median	95% CI
ADA	19	−4.24	−5.30–−3.19	2.19	−4.55	−5.83–−2.39
JAKis	21	−6.14	−6.82–−5.46	1.49	−6.40	−7.20–−5.64
Average rank of first group = 26.18						
Average rank of second group = 15.35						
Mann–Whitney U = 91.50						
Two-tailed probability, *p* = 0.0034						
**% Difference Last-First**						
Group	n	mean	95% CI	SD	median	95% CI
ADA	19	−58.52	−72.48–−44.57	28.95	−68.71	−80.76–−30.80
JAKis	21	−76.59	−83.99–−69.18	16.27	−80.50	−87.30–−70.91
Average rank of first group = 24.47						
Average rank of second group = 16.90						
Mann–Whitney U = 124.00						
Two-tailed probability, *p* = 0.0413						
**Area under curve**						
Group	n	mean	95% CI	SD	median	95% CI
ADA	19	41.14	35.17–47.10	12.37	37.53	30.51–50.44
JAKis	21	32.15	27.67–36.63	9.84	29.16	25.74–36.14
Average rank of first group = 25.15						
Average rank of second group = 16.28						
Mann–Whitney U = 111.00						
Two-tailed probability, *p* = 0.0160						

Abbreviations. CI = confidence interval; SD = standard deviation; ADA = adalimumab; JAKis= Janus kinase inhibitors.

**Table 3:** Criterion values and coordinates of the ROC curve for the RAID cut-off point in CDAI REM or LDA definition. .

Criterion	Sensitivity	95% CI	Specificity	95% CI	+LR	−LR
≤1.23	10.53	1.3–33.1	95.00	75.1–99.9	2.11	0.94
≤1.45	26.32	9.1–51.2	95.00	75.1–99.9	5.26	0.78
≤1.54	26.32	9.1–51.2	90.00	68.3–98.8	2.63	0.82
≤1.66	31.58	12.6–56.6	90.00	68.3–98.8	3.16	0.76
≤1.76	36.84	16.3–61.6	85.00	62.1–96.8	2.46	0.74
≤1.85	47.37	24.4–71.1	85.00	62.1–96.8	3.16	0.62
≤1.98	47.37	24.4–71.1	70.00	45.7–88.1	1.58	0.75
≤2.12	63.16	38.4–83.7	70.00	45.7–88.1	2.11	0.53
≤2.79	63.16	38.4–83.7	65.00	40.8–84.6	1.80	0.57
≤3.09	78.95	54.4–93.9	65.00	40.8–84.6	2.26	0.32
≤3.27	78.95	54.4–93.9	60.00	36.1–80.9	1.97	0.35
≤3.59 *	89.47	66.9–98.7	60.00	36.1–80.9	2.24	0.18
≤6.48	94.74	74.0–99.9	10.00	1.2–31.7	1.05	0.53
≤6.59	94.74	74.0–99.9	5.00	0.1–24.9	1.00	1.05
≤7.02	100.00	82.4–100.0	5.00	0.1–24.9	1.05	0.00
Area under the ROC curve (AUC)		0.722	Youden index J		0.494	
Standard error		0.085	Associated criterion		≤3.59	
95% Confidence interval		0.556–0.853	Sensitivity		89.47	
z statistic		2.597	Specificity		60.00	

Abbreviations and legend. ROC = receiver operating characteristic; CI = confidence interval; +LR = positive likelihood ratio; −LR = negative likelihood ratio; * = optimal cut-off point.

## Data Availability

Study data are available upon reasonable request to the corresponding author.

## References

[1] Salaffi F., de Angelis R., Grassi W., MAPPING Study Group (2005). Prevalence of musculoskeletal conditions in an Italian population sample: Results of a regional community-based study. Clin. Exp. Rheumatol..

[2] Smolen J.S., Landewé R.B.M., Bergstra S.A., Kerschbaumer A., Sepriano A., Aletaha D., Caporali R., Edwards C.J., Hyrich K.L., E Pope J. (2023). EULAR recommendations for the management of rheumatoid arthritis with synthetic and biological disease-modifying antirheumatic drugs: 2022 update. Ann. Rheum. Dis..

[3] Kerschbaumer A., Sepriano A., Bergstra S.A., Smolen J.S., van der Heijde D., Caporali R., Edwards C.J., Verschueren P., de Souza S., E Pope J. (2022). Efficacy of synthetic and biological DMARDs: A systematic literature review informing the 2022 update of the EULAR recommendations for the management of rheumatoid arthritis. Ann. Rheum. Dis..

[4] Silvagni E., Sakellariou G., Bortoluzzi A., Giollo A., Ughi N., Vultaggio L., Scirè C.A. (2021). One year in review 2021: Novelties in the treatment of rheumatoid arthritis. Clin. Exp. Rheumatol..

[5] O’shea J.J., Kontzias A., Yamaoka K., Tanaka Y., Laurence A. (2013). Janus kinase inhibitors in autoimmune diseases. Ann. Rheum. Dis. Ann. Rheum. Dis..

[6] Harrington R., Al Nokhatha S.A., Conway R. (2020). JAK Inhibitors in Rheumatoid Arthritis: An Evidence-Based Review on the Emerging Clinical Data. J. Inflamm. Res..

[7] Harrington R., Harkins P., Conway R. (2023). Janus Kinase Inhibitors in Rheumatoid Arthritis: An Update on the Efficacy and Safety of Tofacitinib, Baricitinib and JAKidacitinib. J. Clin. Med..

[8] Smolen J.S., Landewé R.B.M., Bijlsma J.W.J., Burmester G.R., Dougados M., Kerschbaumer A., McInnes I.B., Sepriano A., van Vollenhoven R.F., de Wit M. (2020). EULAR recommendations for the management of rheumatoid arthritis with synthetic and biological disease-modifying antirheumatic drugs: 2019 update. Ann. Rheum. Dis..

[9] Genovese M.C., Fleischmann R., Combe B., Hall S., Rubbert-Roth A., Zhang Y., Zhou Y., Mohamed M.-E.F., Meerwein S., Pangan A.L. (2018). Safety and efficacy of JAKidacitinib in patients with active rheumatoid arthritis refractory to biologic disease-modifying anti-rheumatic drugs (SELECT-BEYOND): A double-blind, randomised controlled phase 3 trial. Lancet.

[10] Genovese M.C., Kalunian K., Gottenberg J.-E. (2019). Effect of Filgotinib vs Placebo on Clinical Response in Patients with Moderate to Severe Rheumatoid Arthritis Refractory to Disease-Modifying Antirheumatic Drug Therapy: The FINCH2 Randomized Clinical Trial. JAMA.

[11] Pincus T., Castrejón I. (2013). Evidence that the strategy is more important than the agent to treat rheumatoid arthritis. Data from clinical trials of combinations of non-biologic DMARDs, with protocol-driven intensification of therapy for tight control or treat-to-target. Bull. Hosp. Jt. Dis..

[12] Schoels M., Knevel R., Aletaha D., Bijlsma J.W.J., Breedveld F.C., Boumpas D.T., Burmester G., Combe B., Cutolo M., Dougados M. (2010). Evidence for treating rheumatoid arthritis to target: Results of a systematic literature search. Ann. Rheum. Dis..

[13] Salaffi F., Carotti M., Ciapetti A., Gasparini S., Filippucci E., Grassi W. (2011). Relationship between time-integrated disease activity estimated by DAS28-CRP and radiographic progression of anatomical damage in patients with early rheumatoid arthritis. BMC Musculoskelet. Disord..

[14] Caporali R., Conti F., Covelli M., Govoni M., Salaffi F., Ventriglia G., Montecucco C. (2014). Treating rheumatoid arthritis to target: An Italian rheumatologists’ survey on the acceptance of the treat-to-target recommendations. Clin. Exp. Rheumatol..

[15] Solomon D.H., Bitton A., Katz J.N., Radner H., Brown E.M., Fraenkel L. (2014). REVIEW: Treat to target in rheumatoid arthritis: Fact, fiction, or hypothesis?. Arthritis Rheumatol..

[16] Figueroa F.E., Braun-Moscovici Y., Khanna D., Voon E., Gallardo L., Luinstra D., Pina X., Henstorf G., Laurence S., Neiman R. (2007). Patient self-administered joint tenderness counts in rheumatoid arthritis are reliable and responsive to changes in disease activity. J. Rheumatol..

[17] Houssien D.A., Stucki G., Scott D.L. (1999). A patient-derived disease activity score can substitute for a physician-derived disease activity score in clinical research. Rheumatology.

[18] Levy G., Cheetham C., Cheatwood A., Burchette R. (2007). Validation of patient-reported joint counts in rheumatoid arthritis and the role of training. J. Rheumatol..

[19] Salaffi F., Gasparini S., Ciapetti A., Gutierrez M., Grassi W. (2013). Usability of an innovative and interactive electronic system for collection of patient-reported data in axial spondyloarthritis: Comparison with the traditional paper-administered format. Rheumatology.

[20] Salaffi F., Gasparini S., Grassi W. (2009). The use of computer touch-screen technology for the collection of patient-reported outcome data in rheumatoid arthritis: Comparison with standardized paper questionnaires. Clin. Exp. Rheumatol..

[21] Salaffi F., Ciapetti A., Gasparini S., Carotti M., Bombardieri S., New Indices Study Group (2012). The comparative responsiveness of the patient self-report questionnaires and composite disease indices for assessing rheumatoid arthritis activity in routine care. Clin. Exp. Rheumatol..

[22] Salaffi F., Migliore A., Scarpellini M., Corsaro S.M., Laganà B., Mozzani F., Varcasia G., Pusceddu M., Pomponio G., Romeo N. (2010). Psychometric properties of an index of three patient reported outcome (PRO) measures, termed the CLinical ARthritis Activity (PRO-CLARA) in patients with rheumatoid arthritis. New Indices Study..

[23] Gossec L., Dougados M., Rincheval N., Balanescu A., Boumpas D.T., Canadelo S., Carmona L., Daures J.-P., de Wit M., Dijkmans B.A.C. (2008). Elaboration of the preliminary Rheumatoid Arthritis Impact of Disease (RAID) score: A EULAR initiative. Ann. Rheum. Dis..

[24] Gossec L., Paternotte S., Aanerud G.J., Balanescu A., Boumpas D.T., Carmona L., de Wit M., Dijkmans B.A.C., Dougados M., Englbrecht M. (2011). Finalisation and validation of the rheumatoid arthritis impact of disease score, a patient-derived composite measure of impact of rheumatoid arthritis: a EULAR initiative. Ann. Rheum. Dis..

[25] Heiberg T., Austad C., Kvien T.K., Uhlig T. (2011). Performance of the Rheumatoid Arthritis Impact of Disease (RAID) score in relation to other patient-reported outcomes in a register of patients with rheumatoid arthritis. Ann. Rheum. Dis..

[26] Salaffi F., Di Carlo M., Vojinovic J., Tincani A., Sulli A., Soldano S., Andreoli L., Dall’ara F., Ionescu R., Pašalić K.S. (2018). Validity of the rheumatoid arthritis impact of disease (RAID) score and definition of cut-off points for disease activity states in a population-based European cohort of patients with rheumatoid arthritis. Jt. Bone Spine.

[27] Taylor P.C. (2024). The Rheumatoid Arthritis Impact of Disease (RAID) Score in Telemedicine Management of Rheumatoid Arthritis. J. Rheumatol..

[28] Avouac J., Molto A., Allanore Y. (2024). Evaluation of the Rheumatoid Arthritis Impact of Disease (RAID) Score in Assessing Rheumatoid Arthritis Activity in Teleconsultation. J. Rheumatol..

[29] McDaniel A.M., Benson P.L., Roesener G.H., Martindale J. (2005). An integrated computer-based system to support nicotine dependence treatment in primary care. Nicotine Tob. Res..

[30] Saleh K.J., Radosevich D.M., Kassim R.A., Moussa M., Dykes D., Bottolfson H., Gioe T.J., Robinson H. (2006). Comparison of commonly used orthopaedic outcome measures using palm-top computers and paper surveys. J. Orthop. Res..

[31] Anhøj J., Møldrup C. (2004). Feasibility of collecting diary data from asthma patients through mobile phones and SMS (short message service): Response rate analysis and focus group evaluation from a pilot study. J. Med. Internet Res..

[32] Salaffi F., Farah S., Di Carlo M. (2018). Smartphone applications in the clinical care and management of Rheumatic Diseases. Acta Biomed..

[33] Demiris G., Afrin L.B., Speedie S., Courtney K.L., Sondhi M., Vimarlund V., Lovis C., Goossen W., Lynch C. (2008). Patient-centered Applications: Use of Information Technology to Promote Disease Management and Wellness. A White Paper by the AMIA Knowledge in Motion Working Group. J. Am. Med. Inform. Assoc..

[34] Roine R., Ohinmaa A., Hailey D. (2001). Assessing telemedicine: A systematic review of the literature. Can. Med. Assoc. J..

[35] Bernard L., Valsecchi V., Mura T., Aouinti S., Padern G., Ferreira R., Pastor J., Jorgensen C., Mercier G., Pers Y.M. (2022). Management of patients with rheumatoid arthritis by telemedicine: Connected monitoring. A randomized controlled trial. Jt. Bone Spine.

[36] DelliFraine J.L., Dansky K.H. (2008). Home-based telehealth: A review and meta-analysis. J. Telemed. Telecare.

[37] Bowles K.H., Baugh A.C.M. (2007). Applying research evidence to optimize telehomecare. J. Cardiovasc. Nurs..

[38] Meystre S. (2005). The current state of telemonitoring: A comment on the literature. Telemed. E-Health.

[39] Artinian N.T., Harden J.K., Kronenberg M.W., Wal J.S.V., Daher E., Stephens Q., I Bazzi R. (2003). Pilot study of a Web-based compliance monitoring device for patients with congestive heart failure. Hear. Lung.

[40] Balk A.H., Davidse W., Van Dommelen P., Klaassen E., Caliskan K., van der Burgh P., Leenders C.M. (2008). Tele-guidance of chronic heart failure patients enhances knowledge about the disease. Eur. J. Heart Fail..

[41] Dansky K.H., Vasey J., Bowles K. (2008). Use of telehealth by older adults to manage heart failure. Res. Gerontol. Nurs..

[42] Antonicelli R., Mazzanti I., Abbatecola A.M., Parati G. (2008). Impact of home patient telemonitoring on use of β-blockers in congestive heart failure. Drugs Aging.

[43] DeBusk R.F., Miller N.H., Parker K.M., Bandura A., Kraemer H.C., Cher D.J., West J.A., Fowler M.B., Greenwald G. (2004). Care management for low-risk patients with heart failure: A randomized controlled trial. Ann. Intern. Med..

[44] Laramee A.S., Levinsky S.K., Sargent J., Ross R., Callas P. (2003). Case management in a heterogeneous congestive heart failure population: A randomized controlled trial. Arch. Intern. Med..

[45] Aletaha D., Neogi T., Silman A.J., Funovits J., Felson D.T., Bingham III C.O., Birnbaum N.S., Burmester G.R., Bykerk G.R., Cohen M.D. (2010). 2010 rheumatoid arthritis classification criteria: An American College of Rheumatology/European League Against Rheumatism collaborative initiative. Ann. Rheum. Dis..

[46] Studenic P., Aletaha D., de Wit M., Stamm T.A., Alasti F., Lacaille D., Smolen J.S., Felson D.T. (2023). American College of Rheumatology/EULAR remission criteria for rheumatoid arthritis: 2022 revision. Ann. Rheum. Dis..

[47] van Vollenhoven R., Strand V., Takeuchi T., Chávez N., Walter P.M., Singhal A., Swierkot J., Khan N., Bu X., Li Y. (2024). Upadacitinib monotherapy versus methotrexate monotherapy in patients with rheumatoid arthritis: Efficacy and safety through 5 years in the SELECT-EARLY randomized controlled trial. Arthritis Res. Ther..

[48] Fornaro M., Caporali R., Biggioggero M., Bugatti S., De Stefano L., Cauli A., Congia M., Conti F., Chimenti M.S., Bazzani C. (2024). Effectiveness and safety of filgotinib in rheumatoid arthritis patients: Data from the GISEA registry. Clin. Exp. Rheumatol..

[49] Nash P. (2021). Clinical use of JAK 1 inhibitors for rheumatoid arthritis. Rheumatology.

[50] Taylor P.C., Laedermann C., Alten R., Feist E., Choy E., Haladyj E., De La Torre I., Richette P., Finckh A., Tanaka Y. (2023). A JAK Inhibitor for Treatment of Rheumatoid Arthritis: The Baricitinib Experience. J. Clin. Med..

[51] Bartalesi F., Scirè C., Requena-Méndez A., Abad M.A., Buonfrate D., Caporali R., Conti F., Diaz-Gonzalez F., Fernández-Espartero C., Martinez-Fernandez C. (2017). Recommendations for infectious disease screening in migrants to Western Europe with inflammatory arthropathies before starting biologic agents. Clin. Exp. Rheumatol..

[52] Aletaha D., Smolen J. (2005). The Simplified Disease Activity Index (SDAI) and the Clinical Disease Activity Index (CDAI): A review of their usefulness and validity in rheumatoid arthritis. Clin. Exp. Rheumatol..

[53] Bombardier C., Tugwell P. (1982). A methodological framework to develop and select indices for clinical trials: Statistical and judgmental approaches. J. Rheumatol..

[54] Aletaha D., Wang X., Zhong S., Florentinus S., Monastiriakos K., Smolen J.S. (2020). Differences in disease activity measures in patients with rheumatoid arthritis who achieved DAS, SDAI, or CDAI remission but not Boolean remission. Semin. Arthritis Rheum..

[55] Smolen J.S., Aletaha D. (2014). Scores for all seasons: SDAI and CDAI. Clin. Exp. Rheumatol..

[56] Dougados M., Brault Y., Logeart I., van der Heijde D., Gossec L., Kvien T. (2012). Defining cut-off values for disease activity states and improvement scores for patient-reported outcomes: The example of the Rheumatoid Arthritis Impact of Disease (RAID). Arthritis Res. Ther..

[57] Whalley D., Mckenna S.P., De Jong Z., Van Der Heijde D. (1997). Quality of life in rheumatoid arthritis. Br. J. Rheumatol..

[58] Bass M.J., Buck C., Turner L., Dickie G., Pratt G., Robinson H.C. (1986). The physician’s actions and the outcome of illness in family practice. J. Fam. Pract..

[59] Berkanovic E., Hurwicz M.L., Lachenbruch P.A. (1995). Concordant and discrepant views of patients’ physical functioning. Arthritis Care Res..

[60] Chesney A.P., Brown K.A., Poe C.W., Gary H.E. (1983). Physician-patient agreement on symptoms as a predictor of retention in outpatient care. Hosp. Community Psychiatr..

[61] Starfield B., Steinwachs D., Morris I., Bause G., Siebert S., Westin C. (1979). Patient-doctor agreement about problems needing follow-up visit. JAMA.

[62] Francis V., Korsch B.M., Morris M.J. (1969). Gaps in doctor-patient communication. Patients’ response to medical advice. N. Engl. J. Med..

[63] Weingarten S.R. (1995). A study of patient satisfaction and adherence to preventive care practice guidelines. Am. J. Med..

[64] Salaffi F., Franchignoni F., Giordano A., Ciapetti A., Gasparini S., Ottonello M., on behalf of the “NEW INDICES” Study Group (2013). Classical test theory and Rasch analysis validation of the Recent-Onset Arthritis Disability questionnaire in rheumatoid arthritis patients. Clin. Rheumatol..

[65] Salaffi F., Di Carlo M., Farah S., Marotto D., Atzeni F., Sarzi-Puttini P. (2021). Rheumatoid Arthritis disease activity assessment in routine care: Performance of the most widely used composite disease activity indices and patient-reported outcome measures. Acta Biomed..

[66] Tugwell P., Wells G., Strand V., Maetzel A., Bombardier C., Crawford B., Dorrier C., Thompson A. (2000). Clinical improvement as reflected in measures of function and health-related quality of life following treatment with leflunomide compared with methotrexate in patients with rheumatoid arthritis: Sensitivity and relative efficiency to detect a treatment effect in a twelve-month, placebo-controlled trial. Arthritis Rheum..

[67] Pincus T., Strand V., Koch G., Amara I., Crawford B., Wolfe F., Cohen S., Felson D. (2003). An index of the three core data set patient questionnaire measures distinguishes efficacy of active treatment from that of placebo as effectively as the American College of Rheumatology 20% response criteria (ACR20) or the Disease Activity Score (DAS) in a rheumatoid arthritis clinical trial. Arthritis Rheum..

[68] Strand V., Cohen S., Crawford B., Smolen J.S., Scott D.L., for the Leflunomide Investigators Groups (2004). Patient-reported outcomes better discriminate active treatment from placebo in randomized controlled trials in rheumatoid arthritis. Rheumatology.

[69] Wagner E.H., Austin B.T., Davis C., Hindmarsh M., Schaefer J., Bonomi A. (2001). Improving Chronic Illness Care: Translating Evidence Into Action. Health Aff..

[70] Hormaza-Jaramillo A., Arredondo A., Forero E., Herrera S., Ochoa C., Arbeláez-Cortés Á., Aldana A.R.F., Rodriguez A., Amador L., Castaño N. (2022). Effectiveness of Telemedicine Compared with Standard Care for Patients with Rheumatic Diseases: A Systematic Review. Telemed. J. E Health.

[71] Elwyn G., Edwards A., Kinnersley P., Grol R. (2000). Shared decision-making and the concept of equipoise: The competences of involving patients in healthcare choices. Br. J. Gen. Pract..

[72] Wagner E.H., Barrett P., Barry M.J., Barlow W., Fowler Jr F.J. (1995). The effect of a Shared Decision-making Program on rates of surgery for benign prostatic hyperplasia. Pilot results. Med. Care.

[73] Paré G., Jaana M., Sicotte C. (2007). Systematic review of home telemonitoring for chronic diseases: The evidence base. J. Am. Med. Inform. Assoc..

[74] Ekeland A.G., Bowes A., Flottorp S. (2010). Effectiveness of telemedicine: A systematic review of reviews. Int. J. Med. Inform..

[75] Rogers E.M. (2003). Diffusion of Innovations.

[76] Watamura S.E., Donzella B., Kertes D.A., Gunnar M.R. (2004). Developmental changes in baseline cortisol activity in early childhood: Relations with napping and effortful control. Dev. Psychobiol..

[77] Fekedulegn D.B., Andrew M.E., Burchfiel C.M., Violanti J.M., Hartley T.A., Charles L.E., Miller D.B. (2007). Area under the curve and other summary indicators of repeated waking cortisol measurements. Psychosom. Med..

[78] Pruessner J.C., Kirschbaum C., Meinlschmid G., Hellhammer D.H. (2003). Two formulas for computation of the area under the curve represent measures of total hormone concentration versus time-dependent change. Psychoneuroendocrinology.

[79] Simon L.S., Taylor P.C., Choy E.H., Sebba A., Quebe A., Knopp K.L., Porreca F. (2021). The Jak/STAT pathway: A focus on pain in rheumatoid arthritis. Semin. Arthritis Rheum..

[80] Salaffi F., Giacobazzi G., Di Carlo M. (2018). Chronic Pain in Inflammatory Arthritis: Mechanisms, Metrology, and Emerging Targets-A Focus on the JAK-STAT Pathway. Pain. Res. Manag..

[81] Salaffi F., Carotti M., Farah S., Ceccarelli L., Giovagnoni A., Di Carlo M. (2022). Early response to JAK inhibitors on central sensitization and pain catastrophizing in patients with active rheumatoid arthritis. Inflammopharmacology.

[82] Sarzi-Puttini P., Pellegrino G., Giorgi V., Bongiovanni S.F., Varrassi G., Di Lascio S., Fornasari D., Sirotti S., Di Carlo M., Salaffi F. (2024). Inflammatory or non-inflammatory pain in inflammatory arthritis—How to differentiate it?. Best Pract. Res. Clin. Rheumatol..

[83] Fautrel B., Zhu B., Taylor P.C., van De Laar M., Emery P., De Leonardis F., Kannowski C.L., Nicolay C., Kadziola Z., De La Torre I. (2020). Comparative effectiveness of improvement in pain and physical function for baricitinib versus adalimumab, tocilizumab and tofacitinib monotherapies in rheumatoid arthritis patients who are naïve to treatment with biologic or conventional synthetic disease-modifying antirheumatic drugs: A matching-adjusted indirect comparison. RMD Open.

[84] Taylor P.C., Lee Y.C., Fleischmann R., Takeuchi T., Perkins E.L., Fautrel B., Zhu B., Quebe A.K., Gaich C.L., Zhang X. (2019). Achieving Pain Control in Rheumatoid Arthritis with Baricitinib or Adalimumab Plus Methotrexate: Results from the RA-BEAM Trial. J. Clin. Med..

[85] Taylor P.C., Alten R., Gracia J.M.Á.., Kaneko Y., Walls C., Quebe A., Jia B., Bello N., Terres J.R., Fleischmann R. (2022). Achieving pain control in early rheumatoid arthritis with baricitinib monotherapy or in combination with methotrexate versus methotrexate monotherapy. RMD Open.

[86] Fautrel B., Kirkham B., Pope J.E., Takeuchi T., Gaich C., Quebe A., Zhu B., de la Torre I., De Leonardis F., Taylor P.C. (2019). Effect of Baricitinib and adalimumab in reducing pain and improving function in patients with rheumatoid arthritis in low disease activity: Exploratory analyses from RA-BEAM. J. Clin. Med..

[87] Kaufman D.R., Patel V.L., Hilliman C., Morin P.C., Pevzner J., Weinstock R.S., Goland R., Shea S., Starren J. (2003). Usability in the real world: Assessing medical information technologies in patients’ homes. J. Biomed. Inform..

[88] Stoop A.P., Van’t Riet A., Berg M. (2004). Using information technology for patient education: Realizing surplus value?. Patient Educ. Couns..

